# Hidden Criticality of Counterion Condensation Near a Charged Cylinder

**DOI:** 10.1038/s41598-017-09974-9

**Published:** 2017-09-05

**Authors:** Minryeong Cha, Juyeon Yi, Yong Woon Kim

**Affiliations:** 1 Graduate School of Nanoscience and Technology, Korea Advanced Institute of Science and Technology, Daejeon 34141, Korea; 2 Department of Physics, Pusan National University, Busan 46241, Korea

## Abstract

Counterion condensation onto a charged cylinder, known as the Manning transition, has received a great deal of attention since it is essential to understand the properties of polyelectrolytes in ionic solutions. However, the current understanding is still far from complete and poses a puzzling question: While the strong-coupling theory valid at large ionic correlations suggests a discontinuous nature of the counterion condensation, the mean-field theory always predicts a continuous transition at the same critical point. This naturally leads to a question how one can reconcile the mean-field theory with the strong-coupling prediction. Here, we study the counterion condensation transition on a charged cylinder via Monte Carlo simulations. Varying the cylinder radius systematically in relation to the system size, we find that in addition to the Manning transition, there exists a novel transition where all counterions are bound to the cylinder and the heat capacity shows a drop at a finite Manning parameter. A finite-size scaling analysis is carried out to confirm the criticality of the complete condensation transition, yielding the same critical exponents with the Manning transition. We show that the existence of the complete condensation is essential to explain how the condensation nature alters from continuous to discontinuous transition.

## Introduction

Counterion condensation is of fundamental importance in understanding static and dynamic properties of various charged polymers in ionic solutions^1–3^. When a charged polymer is very stiff, it can be considered as an infinite charged cylinder in the presence of neutralizing counterions that are confined in a cylindrical cell^4, 5^. A logarithmic electrostatic potential due to an oppositely charged cylinder attracts counterions and competes with the ion confinement entropy also having the similar system size dependence. As a consequence, a characteristic phase transition occurs in the infinite dilution limit^6–9^; above a critical line charge density (or equivalently, below a critical temperature), a finite fraction of counterions remains bound close to the charged cylinder even in the limit of infinite system size^10^. This problem initially posed by Onsager and later elaborated by Manning^6–8^ and Oosawa^9^, is now well recognized as a vital example to exhibit a thermodynamic transition in polyelectrolytes with counterions^11^.

As a representative feature of the Coulomb fluids, the counterion condensation is also a problem of considerable significance in statistical physics^12–14^, not to mention its importance in polymer science and soft matter. Extensive analytic^15–18^ and numerical^19–21^ works have thus been performed to study the various effects such as fluctuations and correlations^22–25^, neglected in the mean-field (MF) Poisson-Boltzmann (PB) equation. In recent studies most relevant to the present work, Naji and Netz studied the criticality of the Manning transition by proposing the counterion localization length as an order parameter^24, 25^. Although the Manning transition is not a critical phenomenon occurring in translationally-invariant bulk systems, they demonstrated through Monte Carlo (MC) simulations that the Manning transition is a continuous transition with a scale invariant property, and obtained the critical exponent associated with the order parameter. They also found that the ionic correlations do not affect the critical temperature predicted by MF theory and the scaling exponents appear to be universal, i.e., independent of correlation strength^24, 25^.

Despite the fact that the counterion condensation has been studied in great detail for a very long time, the following important questions remain to be addressed. According to the strong-coupling (SC) theory by Netz *et al*., the condensed fraction of counterions should abruptly change from zero to one at the critical point, suggesting a discontinuous transition. In contrast, MF theory always predicts a continuous transition at the same critical point. The first question is then, (i) how can the continuous transition in MF prediction alter its nature into the discontinuous transition in the SC theory by Netz *et al*.? One may conjecture that the transition nature is modified by increasing the coupling strength. However, as mentioned above, the criticality of the Manning transition was shown to be in complete accord with MF theory even at considerably large coupling strengths^24–26^. This gives rise to the second question, (ii) why is the criticality of the system independent of the coupling strength?

In this work, we aim to answer the questions using MC simulations. One of our main findings is that in addition to the well-known Manning transition, there exists another continuous transition into a completely condensed state. A finite-size scaling analysis demonstrates that the complete condensation is a critical phenomenon with scale-invariant properties. We find that the existence of the complete condensation is the key to reconcile the continuous condensation transition of MF theory with the discontinuous transition of the SC theory: In the SC limit, the transition point of the complete condensation merges into the Manning transition point to show discontinuous transition. If the two critical points are separated from each other, the Manning transition maintains its criticality predicted by MF theory even at elevated coupling strengths.

## Results

### Counterion density distribution and condensed fraction

We consider a charged cylinder of radius *R* and surface charge density *σ* (or equivalently, line charge density *τ* = 2*πRσ*), which is placed at the origin and confined inside a cylindrical cell of radius *D*. The system is salt-free but contains *N* neutralizing pointlike counterions of valency *q* that are distributed between the two concentric cylinders, *r* $\in$ (*R*, *D*) with *r* being the radial coordinate of counterion. The system is held at thermal equilibrium with temperature *T*. The solvent is considered as a continuous dielectric medium of the dielectric constant *ε*. We neglect the effect of image charges by assuming that the cylinder has the same dielectric constant with the solution. The Hamiltonian of the system then reads (in units of *k*_*B*_ *T*)^24^

1 $$\beta \,{ {\mathcal H} }_{N}=\frac{{\ell }_{B}}{2}\sum _{i\ne j}\,\frac{{q}^{2}}{|{{\rm{x}}}_{i}-{{\rm{x}}}_{j}|}+2\xi \sum _{i=1}^{N}\,\mathrm{ln}\,(\frac{{r}_{i}}{R}),$$

where ${\ell }_{B}={e}^{2}/\varepsilon {k}_{B}T$ is the Bjerrum length. The Manning parameter, $\xi =q{\ell }_{B}\tau$, corresponds to the rescaled line charge density (or equivalently, to the rescaled inverse temperature) and measures the attractive potential strength by the bare cylinder. In order to mimic an infinitely long cylinder and to minimize artificial finite size effects, we perform MC simulations with periodic boundary conditions along the cylinder axis (*z* direction): The central simulation box of height *H* and with *N* counterions are replicated infinitely many times along the *z*-direction. The long-range Coulomb interactions between counterions in such a periodic system should be counted through the summation over all periodic images, for which we employ the Lekner-Sperb scheme that makes the summation of Coulomb interactions rapidly convergent. Details of the simulation methods can be found elsewhere^24, 25^. The lateral extension parameter, Δ = ln (*D*/*R*), is taken to be a large value to examine the criticality of the condensation phenomena in the infinite dilution limit (Δ → ∞). For an efficient phase space sampling, we employ not only the centrifugal sampling^24^ but also a large scale global move interchanging condensed and unbound counterions, which is important in achieving an equilibration process that is independent of the initial conditions^21^.

**Figure 1:**
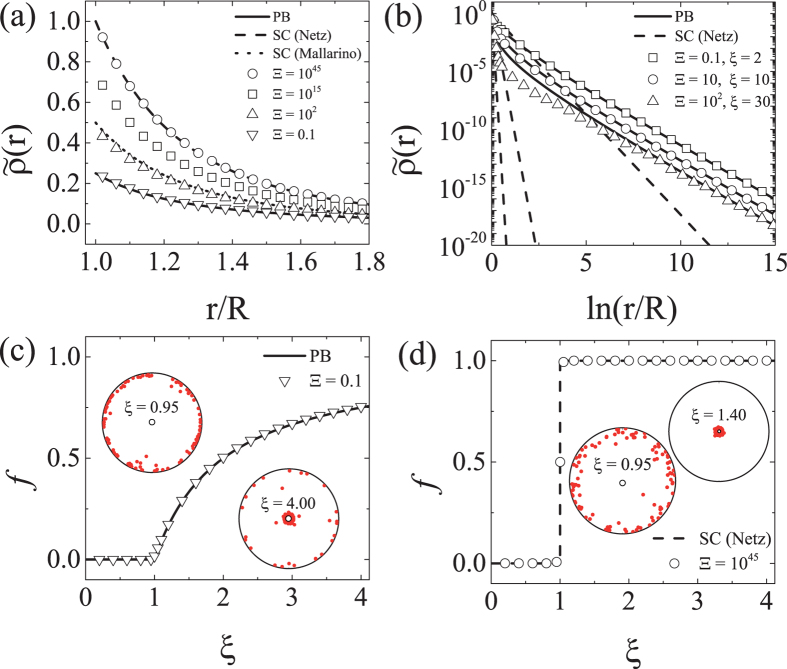
(**a**) Density profiles of counterions for various coupling parameters Ξ, are obtained at the Manning parameter *ξ* = 2 as a function of the radial distance from the charged cylinder axis. (**b**) Density profiles of counterions at greater values of *ξ* when ${\rm{\Xi }}/{\xi }^{2}\sim 0.1$. Solid (dashed) lines represent the PB (SC) theory for the corresponding values of *ξ*. The lower two panels display the condensed fractions as a function of *ξ* (**c**) for a weakly coupled system (Ξ = 0.1) and (**d**) for a strongly coupled system (Ξ = 10^45^). MC simulations are performed for a given lateral parameter Δ = 100 with *N* = 100 counterions. For both cases (**c**,**d**), the simulation data (symbols) are well fitted by the PB and SC theory obtained in the limit Δ → ∞. The circular images are the top views of simulation snapshots of ion distribution at the noted values of *ξ*.

In Fig. 1(a), we present the radial distribution function $\tilde{\rho }(r)=\rho (r)/2\pi {\ell }_{B}{\sigma }^{2}$ of counterions for various values of the coupling parameters ${\rm{\Xi }}=2\pi {q}^{3}{\ell }_{B}^{2}\sigma$ ^27^ at *ξ* = 2. It is well known that for planar geometries, the coupling parameter Ξ measures deviations from MF theory^27–30^. For Ξ = 0.1 and 10^45^, the MC results (symbols) compare well with MF theory (solid line) and SC theory (dashed line), respectively. Recently, Mallarino *et al*. put forward a variation of SC theory by taking account of the small displacement of counterions from their the ground-state configuration in the so-called needle limit^21^. An additional normalization factor is introduced in order to account for the evaporation of counterions^21^, which was further elaborated in a later study^31^. According to their theory, the radial distribution of counterions is determined as $\tilde{\rho }(r)=\mathrm{2(1}-1/\xi {)}^{2}\,{(R/r)}^{2\xi }$ (see Eq. (21) of ref. 21), which is shown to be in a good agreement with our MC data for Ξ = 100 (dotted line).

In Fig. 1(b), we plot the radial density profiles for various values of Ξ with ${{\rm{\Xi }}}^{\mathrm{1/2}}/\xi \sim {\mathscr{O}}\mathrm{(0.1)}$. It shows that the density profiles at longer distance are well described by the PB result even for a relatively large Ξ = 10^2^. We can understand this behavior, considering relevant length scales in the system. Let *a* be the distance between ions when they form a one-dimensional lattice along the charged rod, defined as *a* = *q*/*τ*, and let $\ell$ be the lateral distance between ions on a cylindrical surface as $\sigma {\ell }^{2}\sim q$ (or equivalently, ${\ell }^{2}\sim Ra$). Then one finds that $a/\ell \sim \ell /R\sim {(a/R)}^{\mathrm{1/2}}={{\rm{\Xi }}}^{\mathrm{1/2}}/\xi$. The parameter regime, ${{\rm{\Xi }}}^{\mathrm{1/2}}/\xi \ll 1$ ($a\ll \ell \ll R$), describes the thick cylinder limit where the cylinder radius is much larger than the typical distance between ions along the rod. It is well known in the case of planar surface that at very large distance from the surface, the PB result becomes valid even at large coupling strength^29^. This is because the condensed counterions effectively lowers the coupling strength by screening the surface charges and explains the behavior depicted in Fig. 1(b). In the opposite regime (${{\rm{\Xi }}}^{\mathrm{1/2}}/\xi \gg 1$ or $a\gg \ell \gg R$), the counterions form a quasi one-dimensional lattice on the rod (thin cylinder or needle limit), and the cylinder-ion attraction becomes dominant over the ion-ion repulsion^21^. The condensation behavior in this regime will be examined in detail in the next section.

In order to identify characteristic phases, we consider the condensed fraction *f* as an order parameter^26^, which is defined as the number of counterions residing in a region, *R* ≤ *r* ≤ r_*_:

2 $$f=\frac{2\pi q}{\tau }{\int }_{R}^{{r}_{\ast }}\,dr^{\prime} \,r^{\prime} \,\rho (r^{\prime} ).$$

Based on the inflection-point criterion, r_*_ is chosen to be the inflection point of the accumulated density profile, which is for simplicity approximated in this work as ln (r_*_/*R*) = Δ/2^25^. According to PB equation, a finite fraction of the counterions is condensed if *ξ* > 1, as displayed by the condensed fraction in Fig. 1(c). On the other hand, the SC theory predicts two states, either all counterions are condensed onto the cylinder or totally decondensed [Fig. 1(d)]. The transition occurs also at *ξ* = 1, but it is a discontinuous transition in contrast to the Manning transition. This observation confirms the SC theory by Netz *et al*. which is based on the systematic virial expansion and predicts the condensed fraction of counterions as unity above the critical Manning parameter^27–29^. We note that in order to observe this discontinuous behavior, one has to reach the asymptotically infinite coupling strength, ${\rm{\Xi }}\mathop{ > }\limits_{ \tilde {}}\exp \,({\rm{\Delta }})$ (see below for details). For large but finite Ξ (e.g., Ξ = 10^3^ or 10^4^), the condensed fraction shows a continuous behavior instead of discontinuous one of Fig. 1(d). At large but finite Ξ, according to the theory by Mallarino *et al*., a fraction of condensed ions is assumed to follow the MF theory, and the condensed fraction increases continuously from zero above the critical Manning parameter. We compare their analytic prediction with our numerical results in the next section.

### Complete condensation

The behavior of the counterion condensation is examined in the limit of Δ → ∞, which is indeed realized by an infinite dilution limit (*D* → ∞) and gives *ξ*_1_ = 1 as the critical fixed point of the Manning transition. The condition Δ → ∞ is also met for the line charge limit (*R* → 0). Since only the ratio *D*/*R* enters as a relevant parameter characterizing the system size in both MF and SC theories, the equivalence of the two limits has often been assumed for the condensation phenomenon. However, as mentioned above, *a*/*R* = Ξ/*ξ* ^2^ plays a crucial role in determining the effective counterion configurations on a cylinder: When $a/R\gg 1$, the cylinder-counterion interaction dominates over the ion-ion interactions, validating a single particle picture around a bare charged cylinder. In this respect, the line charge limit (*R* → 0) is not fully equivalent to the infinite dilution limit (*D* → ∞). In order to systematically compare the two limits, we propose a scaling relation, *R*/*a* ~ (*D*/*a*)^−*α*^ so that the two limits can be approached with different rates by controlling *α*. The advantage of employing such a relation becomes more transparent in the following analysis. From now on, we employ *a* as a unit length in MC simulations, while the Gouy-Chapman length, $\mu =1/2\pi q{\ell }_{B}\sigma$, was used as a unit in simulations for Fig. 1.

**Figure 2:**
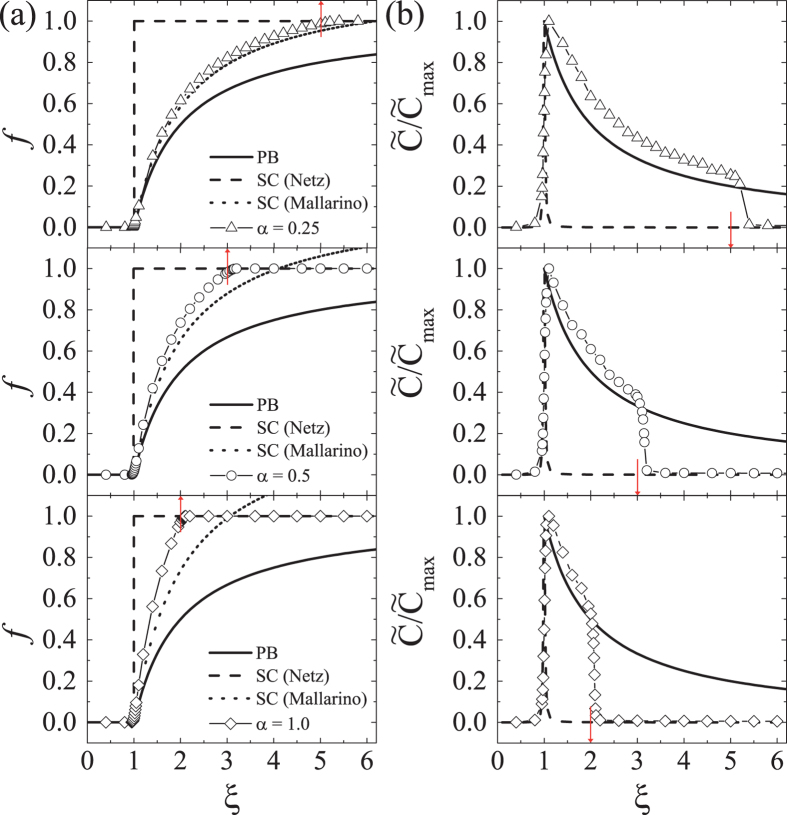
(**a**) Condensed counterion fraction and (**b**) heat capacity are obtained as a function of the Manning parameter *ξ* for various *α*, where the heat capacity is scaled by its maximum value, ${\tilde{C}}_{{\rm{\max }}}$. Simulations are performed with Δ = 100 and *N* = 300 counterions. The marked values by arrows are *ξ* = (1 + *α*)/*α* for each given *α*, at which the condensed fraction is approximately unity, and the heat capacity drops down to zero. The curves are presented for comparison with the PB theory (solid), SC theory by Netz *et al*. (dashed) and SC theory by Mallarino *et al*. (dotted). As *α* increases, the onset point of complete condensation approaches *ξ* = 1, signaling a discontinuous transition in the limit of *α* → ∞.

In Fig. 2(a), we plot the order parameter *f* with increasing *α* while fixing the lateral extension parameter large as Δ = 100, which corresponds to reducing *R* according to ln *R*/*a* = −*α*Δ/(*α* + 1). For comparison, we show in Fig. 2(a) our simulation results together with analytic predictions on the condensed fraction by PB theory (solid), SC theory by Netz *et al*. (dashed) and SC theory by Mallarino *et al*. (dotted, Eq. (70) of ref. 31). As *ξ* varies, the condensed fraction in the considered cases shows three characteristic states, decondensation (*f* = 0), partial condensation (0 < *f* < 1), and complete condensation (*f* = 1). When plotting the condensed fraction according to the SC theory by Mallarino *et al*., one obtains that *f* exceeds unity at elevated *ξ*, which obviously contradicts the physical requirement of charge neutrality and should be considered as an artifact. Our simulation results show that all counterions are unbound, i.e., *f* = 0, for *ξ* < 1, consistent with previous studies, but the complete condensation takes place at *ξ* > *ξ*_2_ with *ξ*_2_ depending on the exponent *α* roughly as

3 $${\xi }_{2}\approx 1+\frac{1}{\alpha }.$$

To discuss the condensation in terms of thermodynamic quantities, we also measure the singular behavior of the dimensionless heat capacity per particle $\tilde{C}=\langle {(\delta  {\mathcal H} )}^{2}\rangle /N$, where $\delta \, {\mathcal H} = {\mathcal H} -\langle  {\mathcal H} \rangle$ [Fig. 2(b)]. The existence of ordered states where all counterions reside either in the outer region *r* > r_*_ (decondensed state) or in the inner region *r* < r_*_ (completely condensed state) is well reflected by $\tilde{C}\approx 0$ either for *ξ* < *ξ*_1_ or for *ξ* > *ξ*_2_. The heat capacity jumps at *ξ*_1_ = 1, signaling the continuous transition, and then abruptly drops at *ξ*_2_, indicating that the system has dual critical points of continuous transition. The locations of the jump and the drop in the heat capacity coincide with the onset points of *f* = 0 and *f* = 1, respectively.

The location of *ξ*_2_ and the presence of the complete condensation can be understood as follows: Suppose that *N* counterions are all bound and form a quasi one-dimensional lattice on the cylinder in a completely condensed state [configuration (a)]^31^. In configuration (b), one of *N* counterions diffuses to the outer boundary. If the free energy of the configuration (a) is lower than that of the configuration (b), the complete condensation can occur. We first consider the Coulomb interaction energy,

4 $$U\equiv \sum _{j}^{\prime} \frac{{q}^{2}{\ell }_{B}}{|{{\rm{x}}}_{i}-{{\rm{x}}}_{j}|}=\frac{\xi }{N}\sum _{j}^{\prime} \frac{1}{\sqrt{{\rho }_{ij}^{2}+{\zeta }_{ij}^{2}}}$$

with *ρ*_*ij*_ = |**r**_*i*_ − **r**_*j*_|/*H* and *ζ*_*ij*_ = (*z*_*j*_ − *z*_*i*_)/*H*, where **r**_*i*_ and *z*_*i*_ denote the position of the *i*th counterion along the radial direction and *z*-axis, respectively. Assume here that the radius of the charged cylinder is much thinner than the average spacing of the counterions when they are equally spaced along the cylinder, that is, $R\ll H/N=q/\tau$. The equality is the charge neutrality condition. Under the assumption, one can estimate the Coulomb energy for the configuration (a), in which the radial distance between counterions is much smaller than the longitudinal distance (${\rho }_{ij}\ll {\zeta }_{ij}$), approximating

5 $${U}^{(a)}\approx 2\frac{\xi }{N}\sum _{\ell =1}\,\frac{N}{\ell },$$

where we let ${\zeta }_{ij}=\ell /N$ and *z*_*i*_ = 0. In the configuration (b), the single counterion is located at the outer boundary (*r*_*i*_ = *D*), and the Coulomb energy due to the other counterions condensed onto the charged cylinder can be approximated as

6 $${U}^{(b)}\approx 2\frac{\xi }{N}\sum _{\ell =1}\,\frac{1}{\sqrt{{(D/H)}^{2}+{(\ell /N)}^{2}}}.$$

Then, the difference between the free energy of the configuration (b) and that of the configuration (a) reads as

7 $$\begin{array}{rcl}{\rm{\Delta }}F & = & {U}^{(a)}-{U}^{(b)}-\mathrm{2(}\xi -\mathrm{1)}\,\mathrm{ln}\,(D/R)\end{array}$$

8 $$\begin{array}{rcl}\quad  & \approx  & \mathrm{2(}\xi -\mathrm{1)}\,\mathrm{ln}\,(D/R)-2\xi \,\mathrm{ln}\,(D/a).\end{array}$$

The second term in Eq. (7) comes from the potential energy by the bare cylinder and a configurational entropy. In obtaining Eq. (8), we used the approximation for $D\tau /q\gg 1$,

9 $$\sum _{\ell =1}\,(\frac{1}{\ell }-\frac{1}{\sqrt{{x}^{2}+{\ell }^{2}}})\approx \,\mathrm{ln}\,x.$$

In the thermodynamic limit (Δ → ∞), the onset point of the complete condensation is determined by ${(\delta F)}_{\xi ={\xi }_{2}}=0$, which leads to

10 $${\xi }_{2}=1-\frac{\mathrm{ln}\,\tilde{D}}{\mathrm{ln}\,\tilde{R}},$$

where the scaled lengths are $\tilde{R}=R\tau /q$ and $\tilde{D}=D\tau /q$. Note that under the conditions, $R\ll H/N=q/\tau$ and $D\gg q/\tau$, used in the derivation of Eq. (10), *ξ*_2_ is not less than unity. Equation (10) indicates that the complete condensation point is determined by the ratio between the logarithmic scales. Therefore, introducing a parameterization, $\tilde{R}=c{\tilde{D}}^{-\alpha }$ with *c* being a finite constant, we find that the onset point of the complete condensation in the large $\tilde{D}$ limit is given by Eq. (3).

Several remarks follow from Eq. (3), or equivalently from Eq. (10) with the parametrization of the logarithmic radii, $\mathrm{ln}\,(\tilde{R})=-\alpha \,\mathrm{ln}\,\tilde{D}$ apart from a small constant *c*. For a finite *α* (0 < *α* < ∞) where *R* → 0 with *D* → ∞, the complete condensation transition occurs at a finite *ξ*_2_ as re-entering the ordered state. For a vanishingly small *α*, i.e., *α* → 0, *R* remains finite even in the limit of *D* → ∞, and the second transition point becomes *ξ*_2_ → ∞. This indicates that the complete condensation for a finite radius (or a finite coupling strength) exists in the limit of *ξ* → ∞, or equivalently in the zero-temperature limit. The condensation behavior is then governed by MF theory predicting only the Manning condensation at *ξ* = 1, as reflected in Fig. 1(c). When *α* → ∞, *R* → 0 even for a large but finite *D*, and the second transition point merges into the Manning transition point as *ξ*_2_ → *ξ*_1_. The heat capacity then develops an asymptotically diverging peak at *ξ* = 1, signaling a discontinuous transition in agreement with the SC theory. The simulation results suggest that the presence of the second transition bridges the two extreme behaviors of PB and SC theories.

We also note that such a large coupling parameter of Ξ = 10^45^ in Fig. 1(d) was used to reproduce the discontinuous behavior of the SC theory by Netz *et al*., which is attainable only for ${\rm{\Xi }}\mathop{ > }\limits_{ \tilde {}}\exp \,({\rm{\Delta }})$, and is not necessary to observe the complete condensation: the coupling parameter is given by Ξ/*ξ* ^2^ = *a*/*R*, and it is in turn related to *α* through ln *R*/*a* = −*α*Δ/(*α* + 1). At the second transition point, *ξ* = *ξ*_2_ ≈ 1 + 1/*α*, of the complete condensation, one can write the coupling parameter as

11 $${\rm{\Xi }}\approx {\xi }_{2}^{2}\,\exp \,({\rm{\Delta }}/{\xi }_{2}).$$

The original SC limit shown in Fig. 1(d) corresponds to the limit of *α* → ∞ or equivalently, *ξ*_2_ → 1. It is clearly seen in Fig. 2 that the complete condensation occurs at much larger value of *ξ*, or equivalently, at much smaller value of Ξ. For example, when the second transition occurs around at *ξ*_2_ ≈ 5 (top in Fig. 2), the coupling parameter becomes Ξ ≈ 10^10^ for Δ = 100. The coupling parameter can be further down to ${\rm{\Xi }}\sim {10}^{4}$ for the second transition occurring at ${\xi }_{2}\sim 20$, which is not far distinct from what one would obtain by considering DNA in the presence of spermines (*q* = 4).

### Criticality: Finite-size scaling analysis

In order to verify the criticality from finite-sized MC simulations, we now turn to the finite-size scaling analysis of the order parameter. We take, for example, *α* = 1 and perform the finite-size scaling upon varying the lateral extension parameter Δ. In analyzing the Manning transition (near *ξ* = *ξ*_1_), we choose *f* ≡ *m*_1_ as an order parameter, and for the complete condensation transition near *ξ* = *ξ*_2_, the scaling behavior of 1 − *f* ≡ *m*_2_ is examined. According to the scaling hypothesis, the order parameter should exhibit a homogeneous-scale invariant property and a power-law decay near the critical point. Since Δ is the only characteristic length scale of the system, the behavior of order parameter with different system sizes satisfy the scaling form in the neighborhood of the critical point as ${m}_{k}={{\rm{\Delta }}}^{-\beta /\nu }{\tilde{m}}_{k}\,({{\rm{\Delta }}}^{\mathrm{1/}\nu }\,{\zeta }_{k})$ with *k* = 1, 2 where *ζ*_*k*_ ≡ 1 − *ξ*_*k*_/*ξ* is the reduced Manning parameter. Here, $\tilde{m}$ is a scaling function that depends only upon the scaling variable, Δ^1/*ν*^ *ζ*. This scaling equation indicates that when estimated from MC simulations for finite size systems with varying lateral extension parameters close to the transition point, the numerical data of the order parameter would fall into a single curve if the correct values of *β* and *ν* are employed.

**Figure 3:**
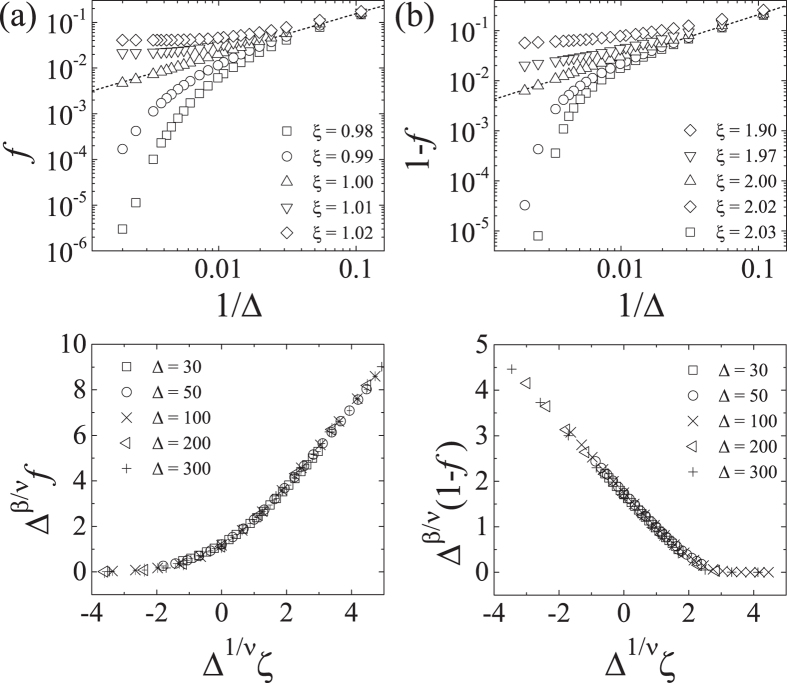
Scaling behaviors of the order parameter *f* near (**a**) *ξ*_1_ = 1 and (**b**) *ξ*_2_ = 2 for *α* = 1. We obtain the condensed fraction, varying the lateral extension parameter Δ with *N* = 300, and choose order parameters, *f* ≡ *m*_1_ for the decondensation transition at *ξ* = *ξ*_1_ and 1 − *f* ≡ *m*_2_ for the complete condensation at *ξ* = *ξ*_2_. The order parameters exhibit a power-law behavior near the critical points, *m*_*k*_ ~ Δ^−*β*/*ν*^, where the exponent is given by *β*/*ν* = 0.88 ± 0.01 for both *k* = 1 and *k* = 2 (these scaling behaviors are presented by the dashed lines). It is also confirmed that the order parameters satisfy a scaling relation ${{\rm{\Delta }}}^{\beta /\nu }\,{m}_{k}={\tilde{m}}_{k}\,({{\rm{\Delta }}}^{\mathrm{1/}\nu }\,{\zeta }_{k})$ with the reduced Manning parameter *ζ*_*k*_ = 1 − *ξ*_*k*_/*ξ*. The scaling exponents are estimated as *β* = 0.98 and *ν* = 1.11 for both *k* = 1 and *k* = 2, which lead to an excellent data collapse onto a single curve.

Figure 3 shows the order parameter as a function of inverse lateral extension parameter 1/Δ for various *ξ* values in the vicinity of the respective transition points. Near *ξ* = *ξ*_1_ and *ξ* = *ξ*_2_, the corresponding order parameter shows a power-law decay *m*_*k*_ ~ Δ^−*β*/*ν*^ with *k* = 1, 2, indicating a scale-invariance property. Here the exponent *β*/*ν* is roughly given by *β*/*ν* ≈ 0.9 both for *m*_1_ of the Manning transition near *ξ* = 1 and for *m*_2_ of the complete condensation transition near *ξ* = 2. Also through a data collapse, we find that a scaling relation, ${m}_{k}={{\rm{\Delta }}}^{-\beta /\nu }\,{\tilde{m}}_{k}\,({{\rm{\Delta }}}^{\mathrm{1/}\nu }\,{\zeta }_{k})$ with a scaling function ${\tilde{m}}_{k}$ and the reduced Manning parameter *ζ*_*k*_ ≡ 1 − *ξ*_*k*_/*ξ*, holds, giving the critical exponent *ν* ≈ 1.1 and *β* ≈ 1.0. Our numerical results clearly indicate that the complete condensation around at *ξ* = *ξ*_2_ is a critical phenomenon, and its critical exponents are shown identical, within our numerical accuracy, with those of the Manning transition at *ξ* = 1.

## Discussion

We have studied the condensation transition of counterions on a charged cylinder and shown that in addition to the well-known Manning transition, there exists a novel phase transition into a complete condensation state. Based on elaborated finite-size scaling analysis, we have also shown that the criticality of the complete condensation is identical to that of the Manning transition, i.e., the complete condensation turns out to have the same critical exponent with the Manning transition. It would be challenging to systematically vary *R*/*a* and to realize the line charge limit in experiments, but our study is of considerable theoretical interest to reveal the hidden criticality of counterion condensations that has been studied for several decades. Furthermore, systematically varying the cylinder radius relative to the system size, we have recovered the MF and SC behaviors numerically. We have also shown that the presence of complete condensation is a key to connect the two limiting theories, i.e., how continuous transition of condensation predicted by the MF theory gradually evolves into discontinuous transition by the SC theory. It would be interesting to extend the present study to further explore the effect of internal structures of counterions on the condensation transitions^32^.
